# Differential intratumoral distributions of CD8 and CD163 immune cells as prognostic biomarkers in breast cancer

**DOI:** 10.1186/s40425-017-0240-7

**Published:** 2017-04-18

**Authors:** Sotirios P. Fortis, Michael Sofopoulos, Nectaria N. Sotiriadou, Christoforos Haritos, Christoforos K. Vaxevanis, Eleftheria A. Anastasopoulou, Nicole Janssen, Niki Arnogiannaki, Alexandros Ardavanis, Graham Pawelec, Sonia A. Perez, Constantin N. Baxevanis

**Affiliations:** 1 Cancer Immunology and Immunotherapy Center, Saint Savas Cancer Hospital, Athens, Greece; 2 Pathology Department, Saint Savas Cancer Hospital, Athens, Greece; 3 Center for Medical Research, Eberhard-Karls Universität, Tübingen, Germany; 4 First Medical Oncology Clinic, Saint Savas Cancer Hospital, Athens, Greece

**Keywords:** Breast cancer, Tumor infiltration, CD8, CD163, Immunoscoring, Prognostic signature

## Abstract

**Background:**

Tumor immune cell infiltrates are essential in hindering cancer progression and may complement the TNM classification. CD8+ and CD163+ cells have prognostic impact in breast cancer but their spatial heterogeneity has not been extensively explored in this type of cancer. Here, their potential as prognostic biomarkers was evaluated, depending on their combined densities in the tumor center (TC) and the tumor invasive margin (IM).

**Methods:**

CD8+ and CD163+ cells were quantified by immunohistochemistry of formalin-fixed, paraffin-embedded (FFPE) tumor tissue samples from a cohort totaling 162 patients with histologically-confirmed primary invasive non-metastatic ductal breast cancer diagnosed between 2000 and 2015. Clinical follow-up (median 6.9 years) was available for 97 of these patients.

**Results:**

Differential densities of CD8+ and CD163+ cells in the combined TC and IM compartments (i.e., high(H)/low(L), respectively for CD8+ cells and the reverse L/H combination for CD163+ cells) were found to have significant prognostic value for survival, and allowed better patient stratification than TNM stage, tumor size, lymph node invasion and histological grade. The combined evaluation of CD8+ and CD163+ cell densities jointly in TC and IM further improves prediction of clinical outcomes based on disease-free and overall survival. Patients having the favorable immune signatures had favorable clinical outcomes despite poor clinicopathological parameters.

**Conclusions:**

Given the important roles of CD8+ and CD163+ cells in regulating opposing immune circuits, adding an assessment of their differential densities to the prognostic biomarker armamentarium in breast cancer would be valuable. Larger validation studies are necessary to confirm these findings.

**Trial registrations:**

Study code: IRB-ID 6079/448/10-6-13

Date of approval: 10/06/2013

Retrospective study (2000–2010)

First patient prospectively enrolled 14/2/2014

**Electronic supplementary material:**

The online version of this article (doi:10.1186/s40425-017-0240-7) contains supplementary material, which is available to authorized users.

## Background

Breast cancer (BCa) is the most common malignancy in women worldwide and is the second leading cause of female cancer deaths [1]. Although systemic therapies have increased survival rates for BCa patients, still there is considerable variation in response rates among patients with distinct clinicopathological parameters which encourages the search for novel prognostic factors contributing to the development of novel treatment options across the different molecular subtypes of BCa. Recent advances in the field of oncoimmunology imply that patients’ pre-existing tumor-specific immunity in the form of tumor-infiltrating lymphocytes (TILs) has a substantial effect on disease progression, thus functioning as potential prognostic biomarkers [2]. Particularly, in colorectal cancer, the type, density and location of TILs (i.e., the “immunoscore”) has been proposed as a more reliable prognostic biomarker than the standard AJCC/UICC TNM-classification [3]. Moreover, results from clinical trials have reported that a robust “immunoscore” predicts responses to therapies, which suggests that the adaptive immune response intratumorally may also function as a predictive biomarker [4].

CD8+ T cells comprise an essential component of the cellular immune system and are indispensable for cell-mediated antitumor immune responses. The presence of CD8+ T-cells in the tumor microenvironment of BCa patients is associated with favorable outcomes in certain molecular subtypes [5]. CD8 is also expressed on a subset of NK cells [6] and a small subpopulation of iNKT cells [7], although their possible presence among CD8+ TILs has not been evaluated. CD163 is a scavenger receptor upregulated by tumor-associated M2 macrophages in an anti-inflammatory tumor microenvironment. CD163 has also been detected in some cancer cells; however these cells are considered as the result of fusion between macrophages and cancer cells [8, 9]. In human malignancies [10], also including BCa [11], the presence of CD163+ M2 macrophages in the tumor stroma correlated with poor overall survival, while contrasting data have been reported for colorectal cancer [12]. In a recent study, localization of CD163+ cells in the tumor stroma, but not the tumor nest was shown to be of clinical relevance for patients with BCa [13]. In contrast, there are studies of BCa patients with different molecular subtypes showing that both stromal as well as intratumoral TILs are equally predictive for clinical outcome [14, 15].

Notwithstanding the general consensus that TILs have a prognostic value in BCa, there remain several issues which hinder their broad application as biomarkers in the routine setting. These include the identification of immune cell populations with the most clinical relevance, their distribution in specific tumor regions and the mode of their evaluation (separate or combined) [16, 17]. Spatial distribution of immune cells in the tumor microenvironment is clinically important, as not only their densities and functions, but also their localization in the different tumor compartments has been associated with clinical outcome [18–21]. Moreover, tumor infiltration by immune cells is a dynamic process with TILs migrating to distinct tumor areas depending on tumor growth properties and factors released by the tumor and cells of the tumor stroma. This heterogeneity suggests that their separate and combined evaluation in well-defined tumor regions would be valuable [14, 20]. To this end, Pagès et al. [22] reported that a combined assessment of the memory (CD45RO+) and cytotoxic phenotypes in TC and IM could increase the accuracy of prediction of clinical outcome for different patient groups in colorectal cancer. However, in evaluations of immune infiltration in BCa, to the best our knowledge there have been no reports on the differential distribution of immune cells in these tumor compartments and their combined evaluation as reliable prognostic/predictive biomarkers. Most recently, Miyan et al. [23] developed a scoring system based on the differential densities of CD3^+^ and CD8^+^ in the TC and IM with the aim of distinguishing between different molecular subtypes of BCa, but they did not address the clinical relevance of such an immunoscore.

In the present study, we aimed to evaluate whether assessment of CD8+ and CD163+ cell densities in single or combined tumor regions (TC and IM) improves the prognostic value of immunoscoring in BCa and allows refinement of conventional prognostic parameters.

## Methods

### Patient selection and characteristics

A total of 162 tissue samples were available from women with histologically-confirmed invasive BCa, diagnosed between 2000 and 2015. Patients with invasive ductal carcinoma were enrolled; patients with tumors directly extending to the chest wall and/or to the skin (ulceration or skin nodules), metastatic disease or bilateral BCa at diagnosis, prior history of any kind of malignant tumor, treatment with any type of neoadjuvant therapy, were excluded from the study. The study was approved by the Institutional Review Board of St. Savas Cancer Hospital (IRB-ID 6079/448/10-6-13).

### Assessment of tumor-infiltrating leukocytes

Formalin-fixed paraffin-embedded (FFPE) tissue blocks were obtained from the archives of the St. Savas Pathology Department. Hematoxylin-Eosin (HE)-stained slides were reviewed by two independent breast pathologists in order to select the most representative slide for each tumor. Sections (4–5 μm) were stained with either CD8 (SP16, 1:80; Thermo Scientific, USA), or CD163 (10D6, 1:400; Biocare). Staining by the antibodies was initially optimized on tonsil tissues (hyperplastic tonsil from tonsillectomy). Immunostaining was performed using the Leica Bond III automation (Leica Biosystems, Germany) and Leica detection kit (Leica Biosystems, Newcastle, UK). The protocol included 30-min high-pH epitope retrieval in the case of CD163 and a low-pH retrieval in the case of CD8, followed by 30 min incubation with the primary antibodies. Reactions were developed with the use of diaminobenzidine (DAB) and sections were counterstained with HE.

**Fig. 1:**
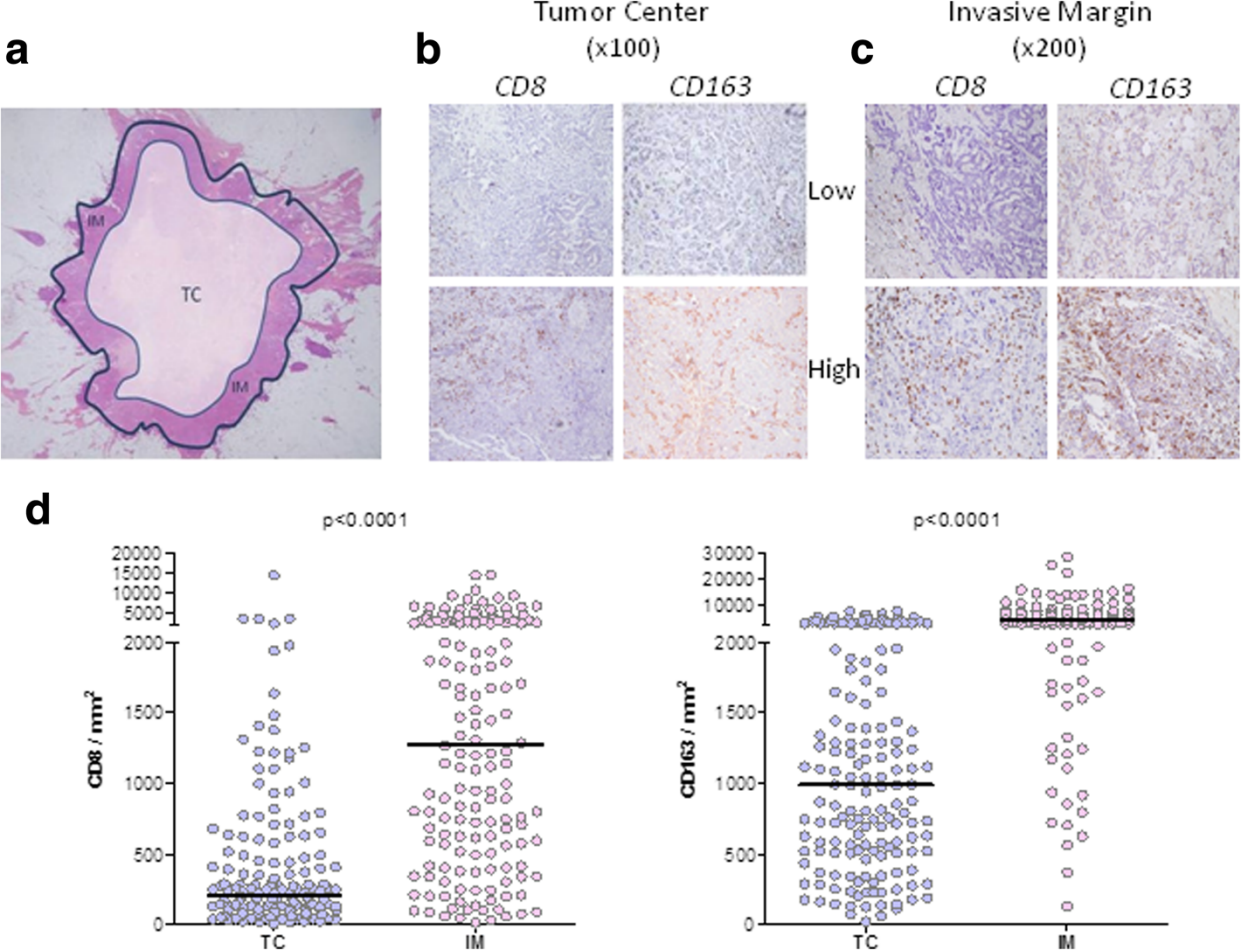
**a** Schematic representation of tumor center (TC) and invasive margin (IM). Representative images of low and high CD8+ and CD163+ cell infiltration densities in TC (**b**) and IM (**c**). **d** CD8+ and CD163+ counts in the tumor center and invasive margin for all patients analyzed (*n* = 162). *Horizontal bars*, median values

Microphotographs from each slide stained with CD8 and CD163 were captured at 12 M resolution (3840 × 3072 pixels) with a Nikon DXM-1200 Digital Eclipse Camera on a Nikon Eclipse E800 microscope with E Plan Achromat Objectives. The software used was Automatic Camera Tamer (ACT-1) Version 2. White balance was calibrated before image capture. The images were saved as JPG at 95% quality without any kind of image processing. Three photographs were captured from the central part (TC) of each tumor at ×100 magnification and three to six at higher magnification (×200), from the tumor infiltrative margins (IM) (Fig. 1a). Representative images are presented in Fig. 1b and c).

The analysis of images and quantification of infiltrating cells was performed using Adobe Photoshop CS6 and ImageJ (http://imagej.nih.gov/ij/). Using Adobe Photoshop CS6 we picked the exact color(s) of positive cells for each marker and then converted the image to black-and-white. The images were analyzed with ImageJ software as the percentage of the surface covered by the specifically stained cells. For each infiltrating subpopulation, visual enumeration in ten representative samples was performed by two independent researchers. Finally, the surface coverage was converted to absolute numbers of infiltrating cells per mm^2^. Median values of infiltrating cells obtained from all TC or IM photos for each patient were recorded and evaluated in further analyses.

### Statistical analysis

Chi-square or Fisher’s exact test and Mann Whitney *t* test statistical analyses were performed by GraphPad Prism v.5.0 software. The same software was used for cumulative survival probabilities testing by Kaplan-Meier analysis with 95% confidence intervals (95%-CIs) and comparison using log rank and Gehan Breslow tests. Hazard ratios were determined using the Cox proportional hazards model. Forward stepwise selection was used in order to exclude less significant covariates, leading to our final model. Multivariate analysis was performed using IBM SPSS statistics 22 software. *P* values <0.05 were considered statistically significant.

## Results

### Patient characteristics

The clinicopathological characteristics of the 162 patients are presented in Table 1. Clinical follow-up data were available for 97 patients (diagnosed from 2000 to 2010), with a median follow-up period of 6.88 years (range: 0.11-10 years). Patients developing loco-regional recurrence or a second primary cancer were excluded from the clinical outcome analyses.

**Table 1:** Clinicopathological characteristics of patients

Total Number of Patients	
*n* = 162	
Median age (years)	Range
54	27–78
Tumor size	n
Tx	1
T1	70
T2	81
T3	10
LN status	n
N0	64
N1	56
N2	32
N3	10
AJCC stage (TNM)	n
I	45
IIA	38
IIB	34
IIIA	34
IIIB	X^a^
IIIC	11
Grade	n
1	4
2	86
3	72
Hormone receptor	n
positive	127
negative	35
HER-2/neu	n
positive	41
negative	121

^a^Stage IIIB patients were not eligible

### Density and intratumoral distribution of CD8+ and CD163+ immune cells in defined tumor regions in BCa patients: correlations with clinicopathological features and clinical outcome

**Fig. 2:**
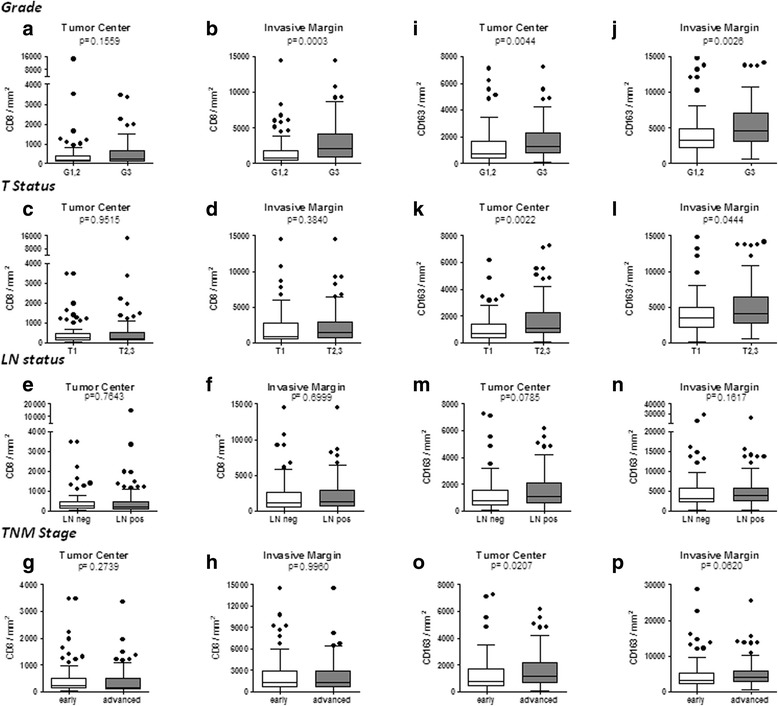
Whisker plots (Tukey) of CD8+ (**a**, **c**, **e**, **g**) and CD163+ (**i**, **k**, **m**, **o**) counts in the tumor center (TC) and CD8+ (**b**, **d**, **f**, **h**) and CD163+ (**j**, **l**, **n**, **p**) counts in the invasive margin (IM) according to clinicopathological variables: Grade (**a**, **b**, **i**, **j**), T status (**c**, **d**, **k**, **l**), LN status (**e**, **f**, **m**, **n**) and TNM stage (**g**, **h**, **o**, **p**). G = grade; T = tumor size; LN = lymph node; TNM stage: early = I&IIA and advanced = IIB&III

Initially, we have counted separately CD8+ and CD163+ cells for both the TC and IM and found significant differences in their absolute numbers distributed within these compartments. On average, the IM contained higher numbers of both cell types than the TC. This was shown for the total patient population (Fig. 1d) as well as for patient subgroups stratified by grade (Fig. 2a, b, i, j), T status (Fig. 2c, d, k, l), node status (Fig. 2e, f, m, n) and TNM pathological stage (Fig. 2g, h, o, p). We also evaluated associations between absolute counts of CD8+ and CD163+ cells in TC and IM regions with the patients’ histological grade, tumor size, lymph node status and pathological stage. The prevalence of CD8+ TILs was higher in poorly-differentiated (histological grade 3) vs grade1,2 tumors both in TC (trend) and IM (highly significant) (Fig. 2a, b). No correlation was found between CD8+ infiltration and T, LN status or disease stage (Fig. 2c-h). Intratumoral CD163+ cell counts in TC and IM were also higher in patients with grade 3 tumors (Fig. 2i, j), with T2,3 stage (Fig. 2k, l) or with positive lymph nodes (Fig. 2m, n) and also in patients with more advanced disease (stages IIB, III) (Fig. 2o, p). These data indicate an association of high absolute numbers of CD163+ cells with a worse patient prognosis.

**Fig. 3:**
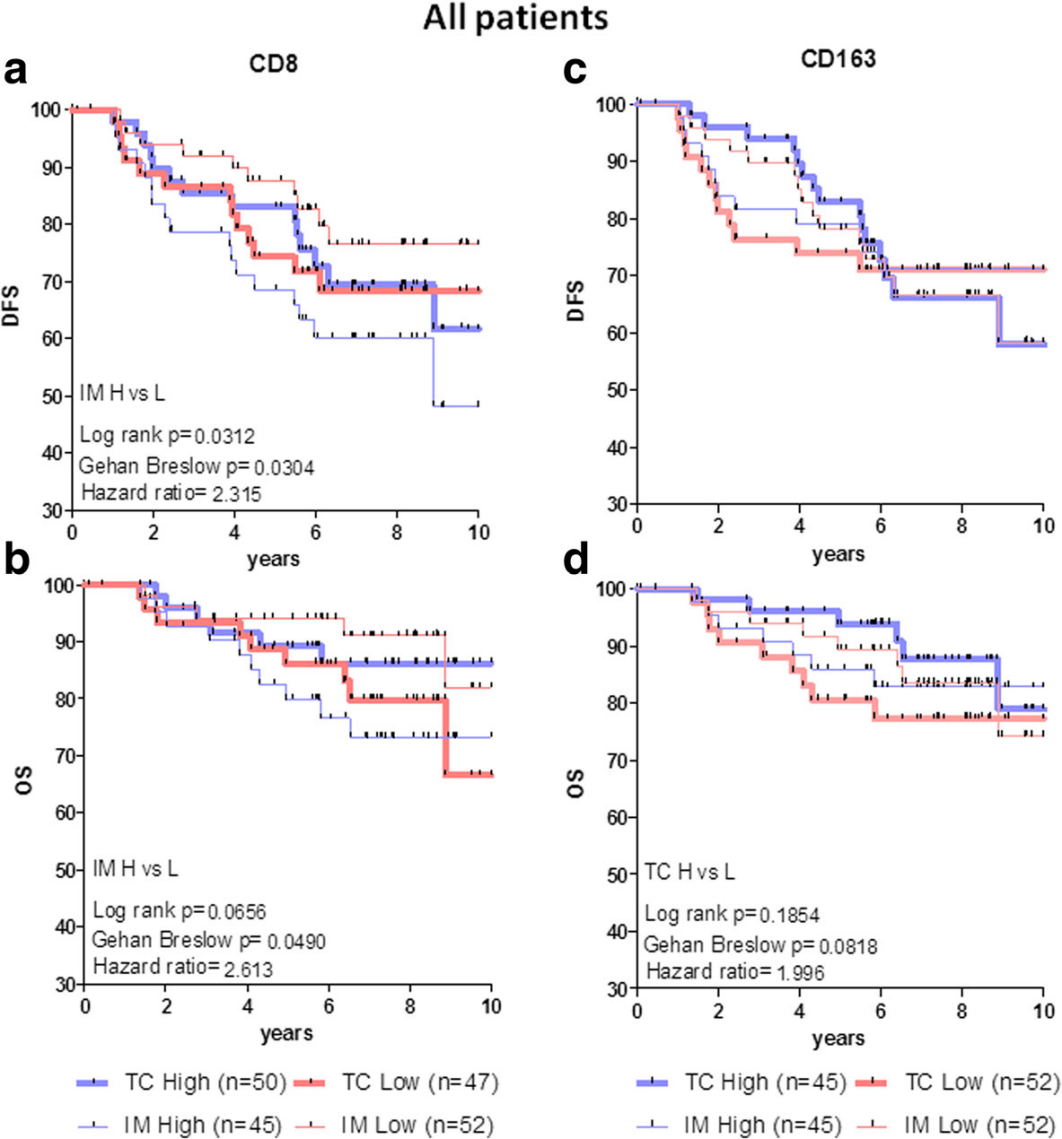
Kaplan-Meier curves illustrating DFS (**a**, **c**) and OS (**b**, **d**) for all patients analyzed according to the density (high = H or low = L) of CD8+ (**a**, **b**) or CD163+ (**c**, **d**) cells in TC or IM. Statistically significant differences or trends and hazard ratios between specific groups are given

Next, we examined the association between CD8 or CD163 densities, separately in TC or IM, with clinical outcomes using the median value to delineate low (L) from high (H) density. Thus, we considered cell densities as L or H when these were below or above the median value for the respective subset from all tissues analyzed. Retrospective analyses in the total patient population with follow-up (*n* = 97), revealed that tumors from patients with longer DFS had significantly lower CD8+ immune cell densities within IM (CD8+ IM L), than tumors from patients who recurred more frequently (Fig. 3a). This latter group had high IM CD8+ cell densities (CD8+ IM H) and significantly reduced DFS (Fig. 3a). In contrast, no significant differences in DFS were found for patients with high or low CD8+ cell densities in the TC (Fig. 3a). There was a strong trend for improved OS in patients with CD8+ IM L vs CD8+ IM H which reached borderline significance by Gehan Breslow analysis (Fig. 3b). CD163+ cell densities in each tumor region (TC or IM) did not allow the stratification of patients into groups with statistically different DFS (Fig. 3c) or OS (Fig. 3d). We also assessed the association of CD8+ and CD163+ cell densities with DFS and OS in subgroups of patients stratified by clinicopathological variables. Kaplan-Meier curves for both DFS and OS showed strong trends for or even significantly better clinical outcomes among groups of patients with favorable vs poor standard clinicopathological parameters (Additional file 1: Figure S1). However, these differences in recurrences and survival were based on the standard TNM staging regardless of the immune cell densities in the different tumor regions, because neither CD8+ nor CD163+ cell densities either in TC or IM had a significant prognostic value when examined separately (Additional file 1: Figures S2–S5). These data suggest that separate analyses of either CD8+ or CD163+ cell densities in single tumor regions are not useful prognostic biomarkers for tumor recurrence and survival in patients with early or advanced BCa.

### Assessment of CD8+ or CD163+ cell densities in the combined tumor regions

**Fig. 4:**
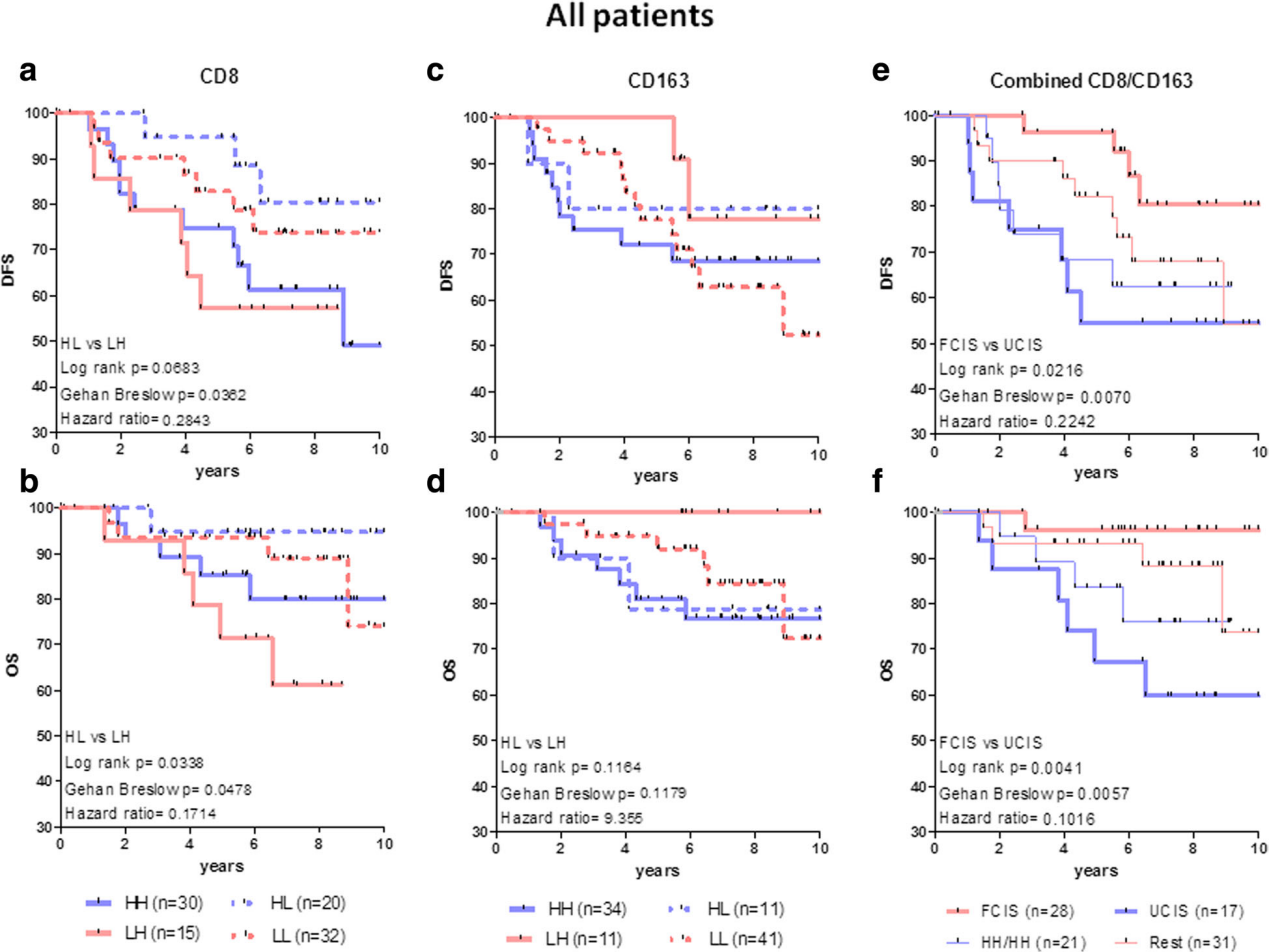
Kaplan-Meier curves illustrating DFS and OS for all patients analyzed according to the density of CD8+ or CD163+ cells in the combined tumor regions (TC and IM) (**a**-**d**). **e**, **f** shows DFS and OS for all patients analyzed according to the combined density of CD8+ and CD163+ cells in the combined tumor regions (TC and IM). For explanation of favorable or unfavorable combined immune signatures (FCIS and UCIS, respectively) see “Results”. HH/HH: high densities for both CD8+ and CD163+ cells jointly analyzed in the combined tumor regions (CT/IM); Rest: LL/LL, HH/LL, and LL/HH. Statistically significant differences and hazard ratios between specific groups are given

Previous reports [18, 22] showed the usefulness of an immune score that is based on the evaluation of TILs in combined tumor regions (TC and IM) for the accurate prediction of tumor recurrence and survival in early stage patients with colorectal cancer. Based on this concept, we investigated whether the analysis of CD8+ and CD163+ cell densities combined in TC and IM could improve the prediction of risk for recurrence or survival also in BCa. For the CD8 marker, the combined analysis of TC plus IM regions with high density in TC and low density in IM (CD8+ HL) versus the reverse combination (low density in TC and high density in IM (CD8+ LH)) allowed a more accurate discrimination for both DFS and OS for the different patient groups; patients with CD8+ HL had a better prognosis than CD8+ LH patients (Fig. 4a, b). In contrast, low CD163+ cell densities in TC combined with high densities in the IM (i.e., CD163+ LH) versus the inverse CD163+ HL, were correlated with strong trends for improved clinical outcome (both DFS and OS; Fig. 4c, d).

### Joint assessment of CD8+ and CD163+ cell densities in the combined tumor regions

The data thus far show that the differential distribution of each immunologic cell marker (CD8+ or CD163+) in the combined tumor regions has a potential prognostic value for both DFS and OS as clinical endpoints. Next, we determined whether a combined evaluation of spatial distribution of CD8+ and CD163+ cell densities in TC and IM could increase the prognostic power for clinical outcome. This type of analyses compared patients with favorable CD8+ HL or CD163+ LH cell densities or both (assigned as the group with the favorable combined immune signatures (FCIS)) and patients having tumors with unfavorable CD8+ LH densities (excluding concomitant CD163+ LH densities) or unfavorable CD163+ HL densities (excluding concomitant CD8+ HL densities), or both. This latter group was assigned as having the unfavorable combined immune signatures (UCIS). We found a more profound discrimination which was highly significant for both DFS and OS among patients having the FCIS versus those who had the UCIS. The estimated 5-year rates for both DFS and OS for patients with the FCIS was 96.3% compared to 54.5% DFS and 67.3% OS rates for those having the UCIS (Fig. 4e, f). Also in this type of analyses, the homogeneous distribution in both tumor regions (i.e., combined CD8+/CD163+ high and/or low cell densities in both TC and IM; HH/HH, LL/LL, HH/LL and LL/HH), could not significantly discriminate for DFS or OS (Fig. 4e, f). In fact, the LL/LL, HH/LL and LL/HH signatures showed a similar trend for improved clinical outcomes, albeit inferior to the FCIS; they were therefore grouped together as the “rest”.

### CD8+ and CD163+ intratumoral cell densities and their correlation with clinical outcome in BCa patients stratified by clinicopathological characteristics

We focused subsequent analyses on CD8+ and CD163+ cells hypothesizing that this intratumoral immune signature could improve the prognostic impact of established clinicopathological parameters. Assuming that the differential densities of CD8+ and CD163+ cells in the combined tumor regions represent different levels of antitumor immunity, we sought to explore whether the favorable signatures, identified in the total patient population, could also have prognostic value for clinical outcome in subgroups of patients who were at high or low risk for recurrence according to standard clinicopathological parameters. For this, we evaluated the prognostic effect of the favorable CD8+ HL and CD163+ LH immune signatures after stratifying the patients by histological grade (Additional file 1: Figure S6A-D), T (Additional file 1: Figure S7A-D) and lymph-node (Additional file 1: Figure S8A-D) status and pathological stage (Additional file 1: Figure S9A-D). Similarly to what was observed for the total patient population, in all stratified groups patients with CD8 HL and patients with CD163 LH, indeed, exhibited better DFS and OS.

**Fig. 5:**
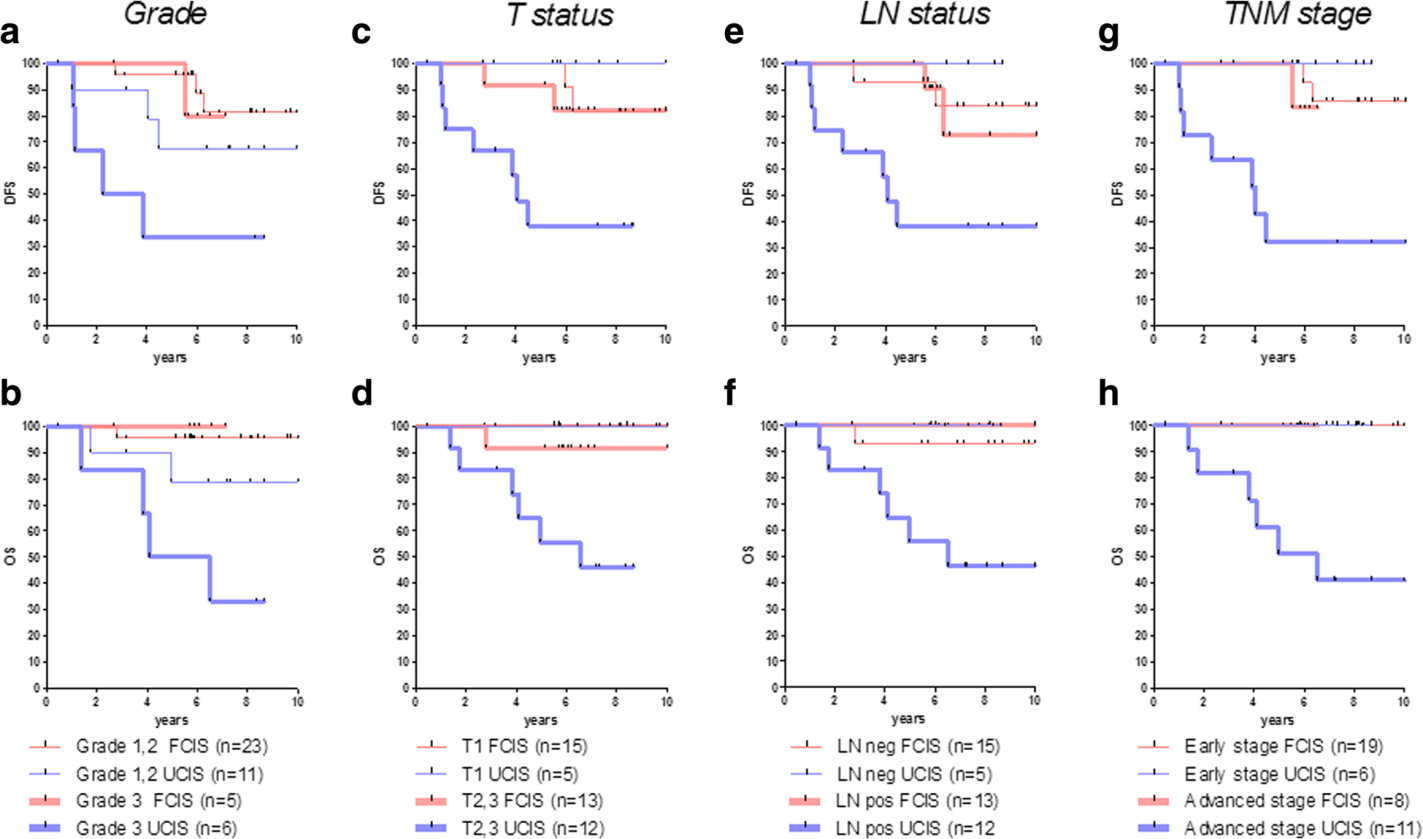
Kaplan-Meier curves comparing DFS and OS for patients with favorable (FCIS) or unfavorable (UCIS) infiltration signatures stratified according to grade (**a**, **b**), T status (**c**, **d**), LN status (**e**, **f**) and TNM stage (**g**, **h**)

Because our results showed a strong association between combined differential densities of CD8+ and CD163+ cells in the tumor compartments TC and IM (i.e., FCIS and UCIS) and clinical outcome in the total patient population (Fig. 4e, f), we also evaluated the prognostic significance of this immune signatures in the same patients stratified by clinicopathological parameters. We found that FCIS strongly correlated with a favorable prognosis regardless of poor standard clinicopathological parameters (Fig. 5a-h). Conversely, UCIS always correlated with a poor prognosis in these groups of patients (Fig. 5a-h and Table 2). Importantly, clinical outcomes (both DFS and OS) for patients with high risk of recurrence having the FCIS were almost indistinguishable from those observed in patients with more favorable clinicopathological parameters (i.e., grade 3 vs 1,2; T2,3 vs T1 status; node positive vs negative; and advanced vs early stage, respectively).

**Table 2:** DFS and OS comparisons in stratified groups of patients with favorable and unfavorable signatures

Groups compared	DFS				OS			
	Hazard Ratio	95% CI of ratio	Log rank p	Gehan Breslow p	Hazard Ratio	95% CI of ratio	Log rank p	Gehan Breslow p
*Grade 3* FCIS vs UCIS	0.2095	0.03457 to 1.270	0.0891	0.0719	0.1438	0.01995 to 1.036	0.0543	0.0589
*FCIS* Grade 3 vs Grade 1,2	1.604	0.1247 to 20.63	0.717	0.6238	0.2931	0.001887 to 45.53	0.6336	0.6336
*T2,3 status* FCIS vs UCIS	0.1924	0.04993 to 0.7417	0.0167	0.0139	0.2015	0.04510 to 0.9000	0.0359	0.0462
*FCIS* T2,3 vs T1	1.684	0.2194 to 12.92	0.6162	0.3775	9.488	0.1837 to 490.0	0.2636	0.2636
*LN pos* FCIS vs UCIS	0.1865	0.04807 to 0.7233	0.0152	0.0054	0.1035	0.02048 to 0.5231	0.0061	0.0070
*FCIS* LN pos vs LN neg	1.375	0.1876 to 10.08	0.754	0.9229	0.1653	0.003201 to 8.537	0.3711	0.3711
*Advanced stage* FCIS vs UCIS	0.1854	0.04531 to 0.7587	0.0191	0.0155	0.1584	0.02981 to 0.8417	0.0306	0.0336
*FCIS* Adv. stage vs Early stage	3.369	0.1545 to 73.43	0.4399	0.2995	0	0 to 0	1	1

Further analyses within each group of patients with worse clinicopathological characteristics revealed that the “rest” (i.e., LL/LL, LL/HH, HH/LL) and HH/HH immune signatures did not have a significant prognostic value for clinical outcome (Additional file 1: Figures S6E, F, S7E, F, S8E, F and S9E, F).

### Inverse relationship between FCIS and state of disease

**Fig. 6:**
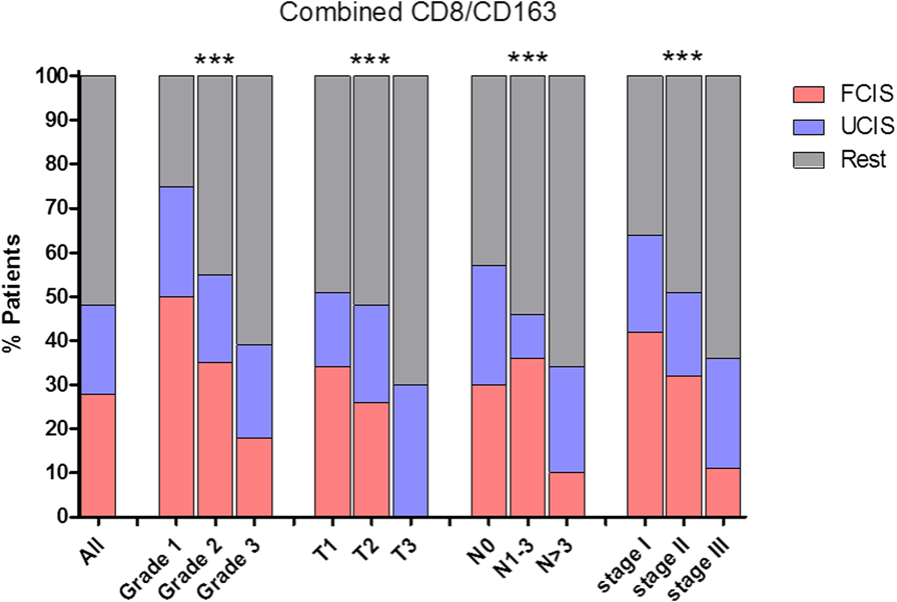
Percentages of total population of patients (all; *n* = 162) or of patients at each histological grade (G1; *n* = 4, G2; *n* = 86,G3; *n* = 72); T status (T1; *n* = 70, T2; *n* = 81,T3; *n* = 10); node status (N0; *n* = 64, N1-3; *n* = 56, *N* > 3; *n* = 42) and pathological stage (stage I; *n* = 45, stage II; *n* = 72, stage III; *n* = 45) expressing FCIS or UCIS or rest (“rest” includes HH/HH; LL/LL; HH/LL and LL/HH)

To better understand the relationship between the in situ immune reaction, as represented by the FCIS and UCIS, with the different histopathologic parameters, we analyzed the percentages of patients expressing these signatures at each histopathologic stage. In this analysis, we included all patients (total *n* = 162). We observed an inverse correlation between the number of patients expressing the FCIS and tumor grade, T and nodal status and TNM stage (Fig. 6). In addition, there was a gradual decrease in the number of FCIS-positive patients from low to high grade tumors, T1 to T3, non-infiltrated with moderate-to-high infiltrated lymph-nodes and early-to-advanced stages (Fig. 6).

### Multivariate analysis of patient DFS and OS according to clinicopathological parameters and combined immune signatures

Results from the multivariate analysis are presented in Table 3. We performed this analysis by initially including into (i) the current, well-established prognostic biomarkers used in breast cancer, i.e., age (patients below or above the 50 year threshold) and pathological parameters, including T status (1,2,3), nodal status (0–3), grade (1,2,3), as well as (ii) HER-2 status (positive or negative), hormone receptor (Estrogen and/or Progesterone) status (positive or negative) and (iii) our immune signatures (FCIS, rest, UCIS, as suggested by the Log-rank survival analyses). T status and hormone receptors status remained significantly associated with both DFS and OS, while grade and HER-2 status were associated only with OS. HER-2 status did not associate with DFS. This result may be explained by the fact that a significant number of patients were diagnosed between 2000 and mid 2006, at which time trastuzumab was not yet the standard-of-care in the adjuvant setting for early stage BCa overexpressing HER-2. Interestingly, despite the small size of samples analyzed, the immune signatures were also significantly associated with DFS and OS. In order to decide on a first model, covariates of lower importance were excluded, using a forward stepwise selection method. As a result, T status, hormone receptors status and our immune signatures were deemed of high importance for DFS and OS.

**Table 3:** Multivariate Cox proportional hazard analysis for DFS and OS of patients with non-metastatic invasive breast cancer

	DFS			OS		
	Hazard Ratio	P	95.0% CI for HR (range)	Hazard Ratio	P	95.0% CI for HR (range)
Model before stepwise selection						
Age^a^	1.031	0.948	0.411–2.589	4.391	0.188	0.486–39.703
T status^b^	2.613	0.010	1.255–5.439	3.679	0.028	1.148–11.793
N stage^b^	1.222	0.512	0.671–2.225	1.125	0.815	0.420–3.010
Grade^b^	1.142	0.750	0.504–2.585	4.189	0.027	1.180–14.867
HER-2/neu	0.928	0.884	0.342–2.520	0.066	0.016	0.007–0.606
Hormone Receptors	0.277	0.004	0.277–0.669	0.168	0.007	0.046–0.621
Signatures^b^	2.063	0.041	1.031–4.126	4.850	0.014	1.374–17.122
Model after stepwise selection						
T status^b^	2.999	0.001	1.602–5.615	3.522	0.005	1.477–8.398
Hormone Receptors	0.269	0.002	0.116–0.621	0.231	0.014	0.072–0.742
Signatures^b^	2.146	0.027	1.091–4.219	4.273	0.006	1.521–11.999
TNM stage^b^	2.180	0.009	1.219–3.898	3.937	0.006	1.494–10.371
Signatures^b^	1.560	0.138	0.866–2.810	2.085	0.091	0.889–4.890

^a^Age under 50 and over 50 years old

^b^All categorical covariates were transformed into numeric codes as follows : T status (T1; 1, T2; 2, T3; 3), N stage (N0; 0, N1; 1, N2; 2, N3; 3), Grade (G1; 1, G2; 2, G3; 3), Signatures (FCIS; 1, Rest; 2, UCIS; 3) TNM stage (I; 1, II; 2, III

## Discussion

In the present study, we demonstrate that the combined evaluation of CD8+ or CD163+ immune cell densities in the tumor center and invasive margin allows better stratification and improves the prognostic value of TNM staging in BCa. Our data also suggest that the combined evaluation of CD8+ and CD163+ cell densities jointly in TC and IM could improve the accuracy of prediction for DFS and OS. Initially, focusing on the most common TIL population, the CD8+ cells, we observed maximum DFS and OS when high CD8+ densities in the TC were combined with low densities in the IM (i.e., HL). In contrast, the inverse combination (i.e., LH) was associated with significantly higher recurrence rates and reduced OS. This finding is challenging given that the most potent favorable immunoscore value in colorectal cancer combines high CD8+ densities both in TC and IM [18, 22]. We also aimed to investigate whether the localization of macrophages in primary BCa could be of clinical relevance. CD163 represents a marker expressed primarily by the anti-inflammatory (M2) subtypes of macrophages [24]. We detected higher numbers of CD163+ than CD8+ cells in both tumor regions, consistent with the increased influx of TAMs in breast tumors [24]. In agreement with a recent report [13], we found that CD163+ cells in TC positively correlated with poor clinicopathological features, emphasizing the importance of analyzing densities of these cells locally as a prognostic factor. However, in line with our CD8+ cell scoring, we found that evaluation of CD163+ cell densities in single regions did not strongly associate with DFS or OS. Nonetheless, combined analyses indicated a favorable clinical outcome in patients having low CD163+ cell densities in TC combined with high densities in the IM. Interestingly, the poorest prognostic impact was noticed among patients whose tumors’ compartments were inversely infiltrated by CD163+ cells, namely with high TC vs low IM densities.

Presently it is not clear what may cause such differential distributions among tumor compartments. Given the interrelationship between tumor biology features and immune reactions we may hypothesize that depending on locally tumor-secreted inflammatory molecules, immune cells may accumulate at distinct areas within the tumor microenvironment. Such a compartmentalization may then influence the functional status of these immune cells as this has been shown for intratumoral DCs with pro- and anti-tumor properties [25–27]. In an analogous fashion, various chemokines have been shown to selectively attract CD8+ cells at different tumor compartments [28–34], suggesting that the location of the various TIL populations is a dynamic process with pro- or antitumor effects, depending on tumor biology reflecting the stage of disease, which associates with clinical outcome in different malignancies, including BCa [23, 25, 35] In the same lines, we show herein that cells with opposing functions (i.e.,CD8+ and CD163+ cells) in distinct tumor regions and at differential densities have significant predictive roles; yet, at present it is not known whether their location in either compartment is due to migratory processes induced by locally secreted factors or architectural (contextual) barriers capturing these cells in the tumor compartments [14].

We also determined that such favorable immune cell differential densities were correlated with improved clinical outcomes in groups of patients with otherwise poor clinicopathological variables (i.e., advanced stage, large tumors, high grade and positive lymph nodes) which were comparable to those for patients with good prognosis according to standard clinicopathological criteria (i.e., early pathological stage, low volume tumors and histologic grade and negative lymph nodes).

From a theoretical point of view, our results suggest that by the time human BCa become clinically detectable (i.e., the escape phase of immunoediting), the adaptive immune response is still active playing a significant role in delaying tumor progression. This may not be quite compatible with the immunoediting theory [36], given that the beneficial effect of adaptive immunity may persist throughout tumor progression, as we show here for BCa patients with more advanced disease having similar DFS and OS with early stage patients provided they have a favorable immune signature. We may propose that our favorable immune signature slows down tumor growth rates, thus increasing OS. At this point we should mention that such pre-existing immunity may, to a certain degree, be directed towards neoantigens expressed by the tumor through the emergence of nonsynonymous somatic mutations [37]. Alternatively, the FCIS may contribute to a modification of tumor stroma and tumor cells in order to negatively influence angiogenesis and extravasation and in this way to influence tumor evolution and progression. Alterations in immune cell densities and function that may occur while disease is progressing could support tumor evasion from immunosurveillance. The underlying mechanisms for the so-called acquired immune resistance have been mostly attributed to epigenetic changes [35, 38] and most recently to upregulation of alternate immune checkpoints [39] as well as to the loss of nonsynonymous somatic mutations which leads to low densities or elimination of neoantigens [40]. However, also in this case the beneficial effect of intratumoral antitumor immunity may persist during tumor progression, attenuating the metastatic potential of the tumor and resulting in better clinical outcomes. Thus, intratumoral immune infiltrates may not only reflect pre-existing immunity but also the immune response to therapy. Therefore, it will be of great importance for decisions making in terms of patients’ clinical management to know whether and to what level the patients’ original immune status influences survival independently of therapy.

Because the primary tumor in our BCa population was removed by surgery, the prognostic value associated with the host intratumoral immune response may reflect the quantity and quality of circulating cytotoxic effectors recognizing and lysing tumor cells in peripheral blood, lymphoid tissues or other anatomical sites. It is, however, at present unknown whether a correlation exists between the immune phenotype of tumors and the responsiveness of peripheral immune cells to immune stimulation. Mortarini et al. [41] observed that the frequency of T regulatory cells at the tumor site, correlated with increased circulating levels of TGFβ and decreased responsiveness to IL-2 stimulation by peripheral T cells. In the same lines, we have demonstrated an inverse correlation between serum TGFβ levels with immunological T cell responses to a HER-2/neu hybrid peptide used to vaccinate prostate cancer patients [42]. This points to the need to test whether there is an interrelation between the status of activation of tumors and the behavior of circulating cells and to compare whether this drives the systemic alteration of immune function associated with the cancer bearing status. Identification of a correlation would allow functional assessment of tumors which consequently would predict prognosis by testing circulating cells. Such analyses may be useful for assessing the role of adaptive immune responses at the tumor site and the periphery as a continuum alongside with tumor evolution.

We also show here that the frequency of patients exhibiting the FCIS was decreased among groups with poor clinicopathological variables suggesting that favorable CD8+ and CD163+ cell densities inversely correlated with the TNM stage. We may propose that disease progression associates with a worsening of this antitumor immune response resulting in a gradually increasing immune escape. As also discussed above,, even at this stage, the strength of the immune response could be an essential parameter for efficiently controlling tumor evolution.

We have shown that the differential densities and spatial distribution of CD8+ and CD163+ cells as described by the FCIS could identify patients with increased DFS or patients who lived longer despite the fact that these were in late tumor stages according to the TNM classification. This indicates that the FCIS, as defined here, constitutes a novel candidate-indicator beyond TNM staging to improve the prediction of clinical outcomes.

## Conclusions

Our study is the first to reveal that differential densities of the same immune cell subpopulation infiltrating the primary tumor at the TC and IM may have opposing prognostic impacts in BCa. The FCIS defined herein predicts favorable clinical outcomes (both DFS and OS) across heterogeneous groups of patients with advanced stage disease, large tumors, invaded lymph nodes and high histological grade tumors, and complements the established robust standard prognostic parameters in BCa. Thus, by associating TNM-based classifications and combined intratumoral immune signatures, as shown here with the FCIS, we may provide preliminary evidence for defining new subgroups of patients with distinct prognosis.

## Additional file

Additional file 1: Figure S1. DFS and OS in patients according to standard clinicopathological variables. **Figures S2**-**S5.** DFS (A, C) and OS (B, D) for patients stratified by grade (Figure S2), T status (Figure S3), nodal status (Figure S4) and pathological TNM stage (Figure S5) and analyzed according to the density of CD8+ (A, B) or CD163+ (C, D) cells in TC or IM. **Figures S6**-**S9.** Kaplan-Meier curves illustrating DFS (A, C, E) and OS (B, D, E) for patients stratified by grade (Figure S6), T status (Figure S7), nodal status (Figure S8) and pathological TNM stage (Figure S9) and analyzed according to the density of CD8+ (A, B) or CD163+ (C, D) cells in the combined tumor regions and to the combined immune signatures (E, F). The statistics with significant differences or strong trends between groups and the corresponding hazard ratios are shown in the respective plots. (PDF 356 kb)

## Abbreviations

BCa: Breast cancer
DCs: Dendritic cells
DFS: Disease free survival
FCIS: Favorable combined immune signature
FFPE: Formalin-fixed, paraffin-embedded
HE: Hematoxylin-eosin
HR: Hazard ratio
IL-2: Interleukin-2
IM: Invasive margin
iNKT: Invariant natural killer T
NK: Natural killer cell
OS: Overall survival
TAMs: Tumor-associated macrophages
TC: Tumor center
TGFβ: Transforming growth factor beta
TILs: Tumor-infiltrating lymphocytes
UCIS: Unfavorable combined immune signature
