# Erratum to: Differential intratumoral distributions of CD8 and CD163 immune cells as prognostic biomarkers in breast cancer

**DOI:** 10.1186/s40425-017-0248-z

**Published:** 2017-05-30

**Authors:** Sotirios P. Fortis, Michael Sofopoulos, Nectaria N. Sotiriadou, Christoforos Haritos, Christoforos K. Vaxevanis, Eleftheria A. Anastasopoulou, Nicole Janssen, Niki Arnogiannaki, Alexandros Ardavanis, Graham Pawelec, Sonia A. Perez, Constantin N. Baxevanis

**Affiliations:** 1 Cancer Immunology and Immunotherapy Center, Saint Savas Cancer Hospital, Athens, Greece; 2 Pathology Department, Saint Savas Cancer Hospital, Athens, Greece; 3 Center for Medical Research, Eberhard-Karls Universität, Tübingen, Germany; 4 First Medical Oncology Clinic, Saint Savas Cancer Hospital, Athens, Greece

## Erratum

After publication of this article [1], it was noticed that Table 3 contained errors introduced during the production process. The corrected Table 3 can be seen below and the original article has been updated to correct the errors.

**Table 3:** Multivariate Cox proportional hazard analysis for DFS and OS of patients with non-mesastatic invasive breast cancer

	DFS			OS		
	Hazard Ratio	*P*	95.0% CI for HR (range)	Hazard Ratio	*P*	95.0% CI for HR (range)
TNM stage^b^	2.180	0.009	1.219–3.898	3.937	0.006	1.494–10.371
Signatures^b^	1.560	0.138	0.866–2.810	2.085	0.091	0.889–4.890
Model before stepwise selection						
Age^a^	1.031	0.948	0.411–2.589	4.391	0.188	0.486–39.703
T status^b^	2.613	0.010	1.255–5.439	3.679	0.028	1.148–11.793
N stage^b^	1.222	0.512	0.671–2.225	1.125	0.815	0.420–3.010
Grade^b^	1.142	0.750	0.504–2.585	4.189	0.027	1.180–14.867
HER-2/neu	0.928	0.884	0.342–2.520	0.066	0.016	0.007–0.066
Hormone Receptors	0.277	0.004	0.277–0.669	0.618	0.007	0.046–0.621
Signatures^b^	2.063	0.041	1.031–4.126	4.850	0.014	1.374–17.122
Model after stepwise selection						
T status^b^	2.999	0.001	1.602–5.615	3.522	0.005	1.477–8.398
Hormone Receptors	0.269	0.002	0.116–0.621	0.231	0.014	0.072–0.742
Signatures^b^	2.146	0.027	1.091–4.219	4.273	0.006	1.521–11.999

^a^Age under 50 and over 50 years old

^b^All categorical covariates were transformed into numeric codes as follows: T status (T1; 1, T2; 2, T3; 3), N stage (NO; 0, N1; 1, N2; 2, N3; 3), Grade (G1; 1, G2; 2, G3; 3), Signatures (FCIS; 1, Rest; 2, UCIS; 3) TNM stage (I; 1, II; 2, III; 3)
