# Supplementary material for: Differential intratumoral distributions of CD8 and CD163 immune cells as prognostic biomarkers in breast cancer
Source: J Immunother Cancer. 2017 Apr 18;5:39. doi: 10.1186/s40425-017-0240-7 (PMC5395775; doi:10.1186/s40425-017-0240-7)
Supplement: Additional file 1: Figure S1. — DFS and OS in patients according to standard clinicopathological variables. Figures S2-S5. DFS (A, C) and OS (B, D) for patients stratified by grade (Figure S2), T status (Figure S3), nodal status (Figure S4) and pathological TNM stage (Figure S5) and analyzed according to the density of CD8+ (A, B) or CD163+ (C, D) cells in TC or IM. Figures S6-S9. Kaplan-Meier curves illustrating DFS (A, C, E) and OS (B, D, E) for patients stratified by grade (Figure S6), T status (Figure S7), nodal status (Figure S8) and pathological TNM stage (Figure S9) and analyzed according to the density of CD8+ (A, B) or CD163+ (C, D) cells in the combined tumor regions and to the combined immune signatures (E, F). The statistics with significant differences or strong trends between groups and the corresponding hazard ratios are shown in the respective plots. (PDF 356 kb) [file 40425_2017_240_MOESM1_ESM.pdf]

Sup. Fig 1

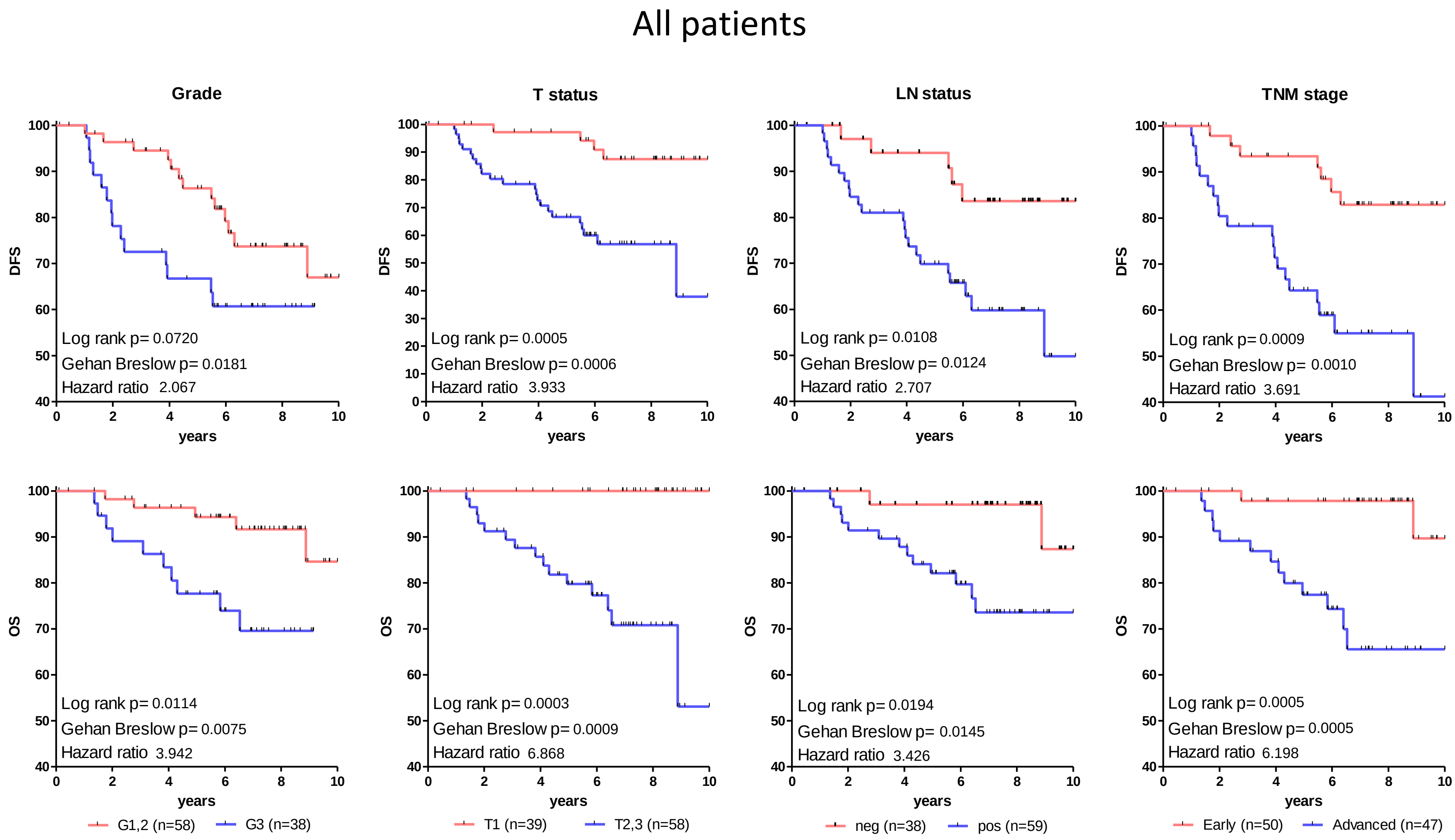

All patients
Grade
T status
LN status
TNM stage
DFS
OS
years
Log rank p= 0.0720
Gehan Breslow p= 0.0181
Hazard ratio 2.067
Log rank p= 0.0005
Gehan Breslow p= 0.0006
Hazard ratio 3.933
Log rank p= 0.0108
Gehan Breslow p= 0.0124
Hazard ratio 2.707
Log rank p= 0.0009
Gehan Breslow p= 0.0010
Hazard ratio 3.691
Log rank p= 0.0114
Gehan Breslow p= 0.0075
Hazard ratio 3.942
Log rank p= 0.0003
Gehan Breslow p= 0.0009
Hazard ratio 6.868
Log rank p= 0.0194
Gehan Breslow p= 0.0145
Hazard ratio 3.426
Log rank p= 0.0005
Gehan Breslow p= 0.0005
Hazard ratio 6.198
G1,2 (n=58)
G3 (n=38)
T1 (n=39)
T2,3 (n=58)
neg (n=38)
pos (n=59)
Early (n=50)
Advanced (n=47)

Sup. Fig. 2

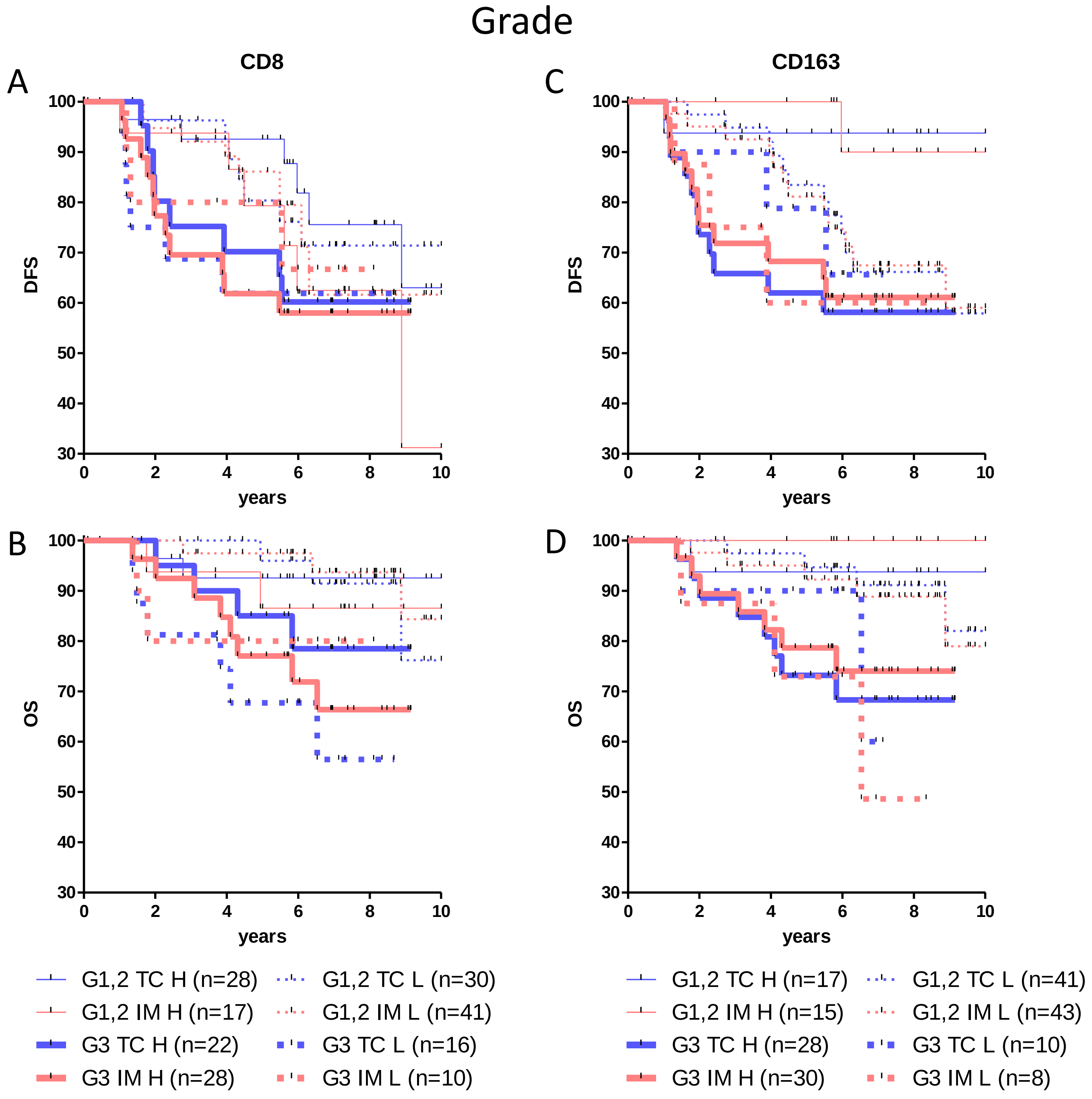
Grade
CD8
CD163
A
B
C
D
DFS
OS
years
100
90
80
70
60
50
40
30
0
2
4
6
8
10
G1,2 TC H (n=28)
G1,2 IM H (n=17)
G3 TC H (n=22)
G3 IM H (n=28)
G1,2 TC L (n=30)
G1,2 IM L (n=41)
G3 TC L (n=16)
G3 IM L (n=10)
G1,2 TC H (n=17)
G1,2 IM H (n=15)
G3 TC H (n=28)
G3 IM H (n=30)
G1,2 TC L (n=41)
G1,2 IM L (n=43)
G3 TC L (n=10)
G3 IM L (n=8)

Sup. Fig. 3

T status

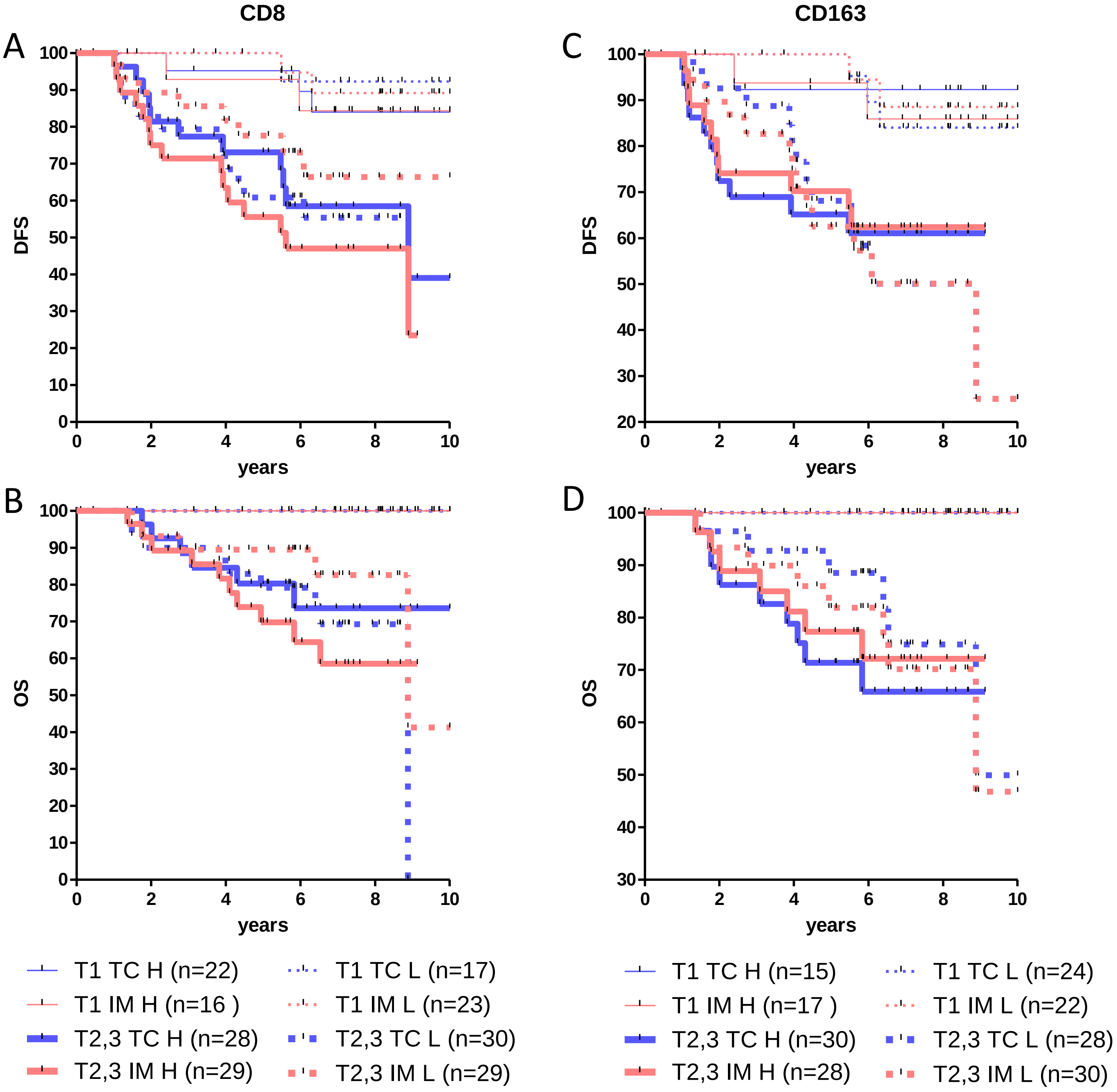

Sup. Fig. 4

LN status

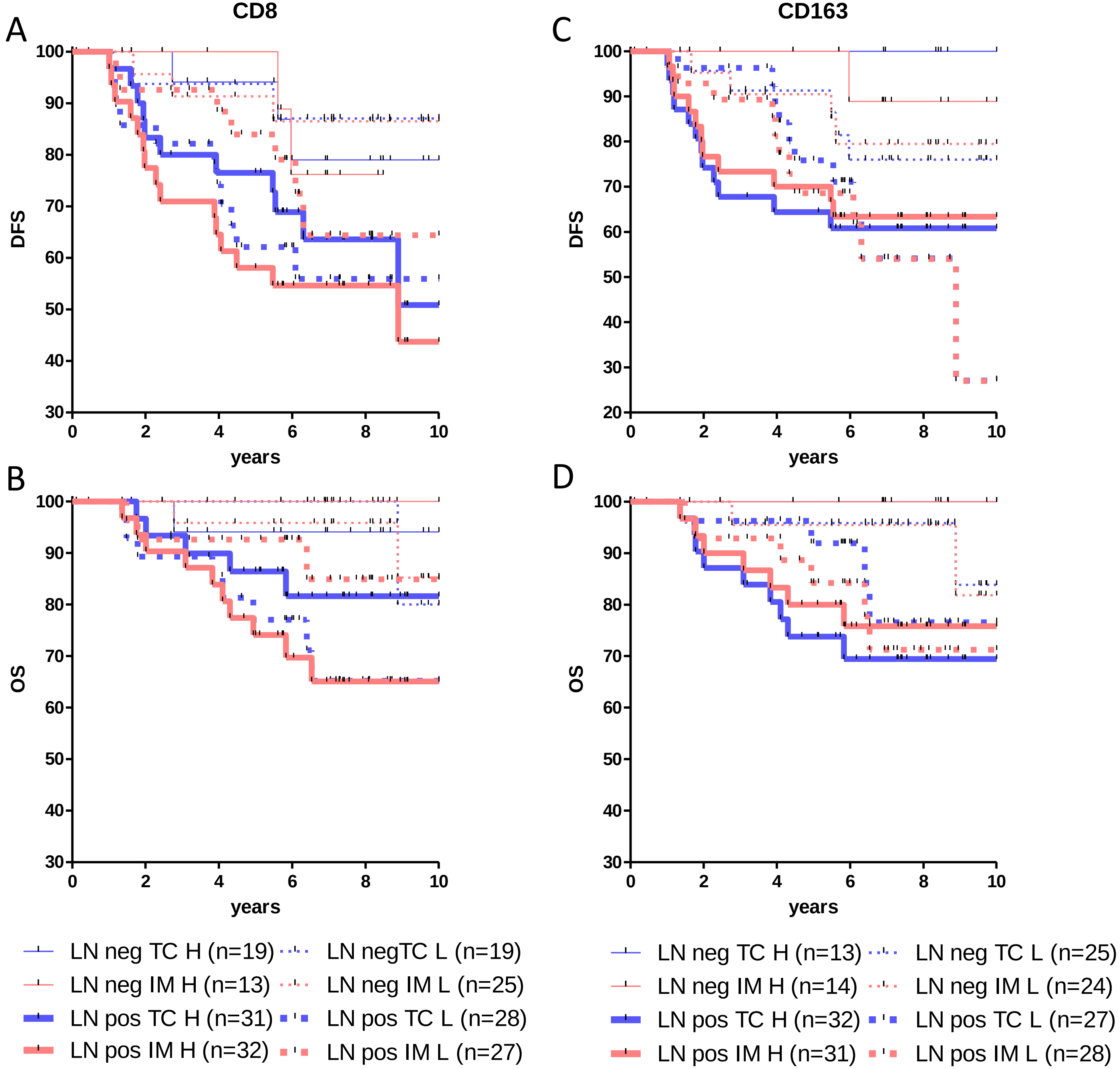

Sup. Fig. 5

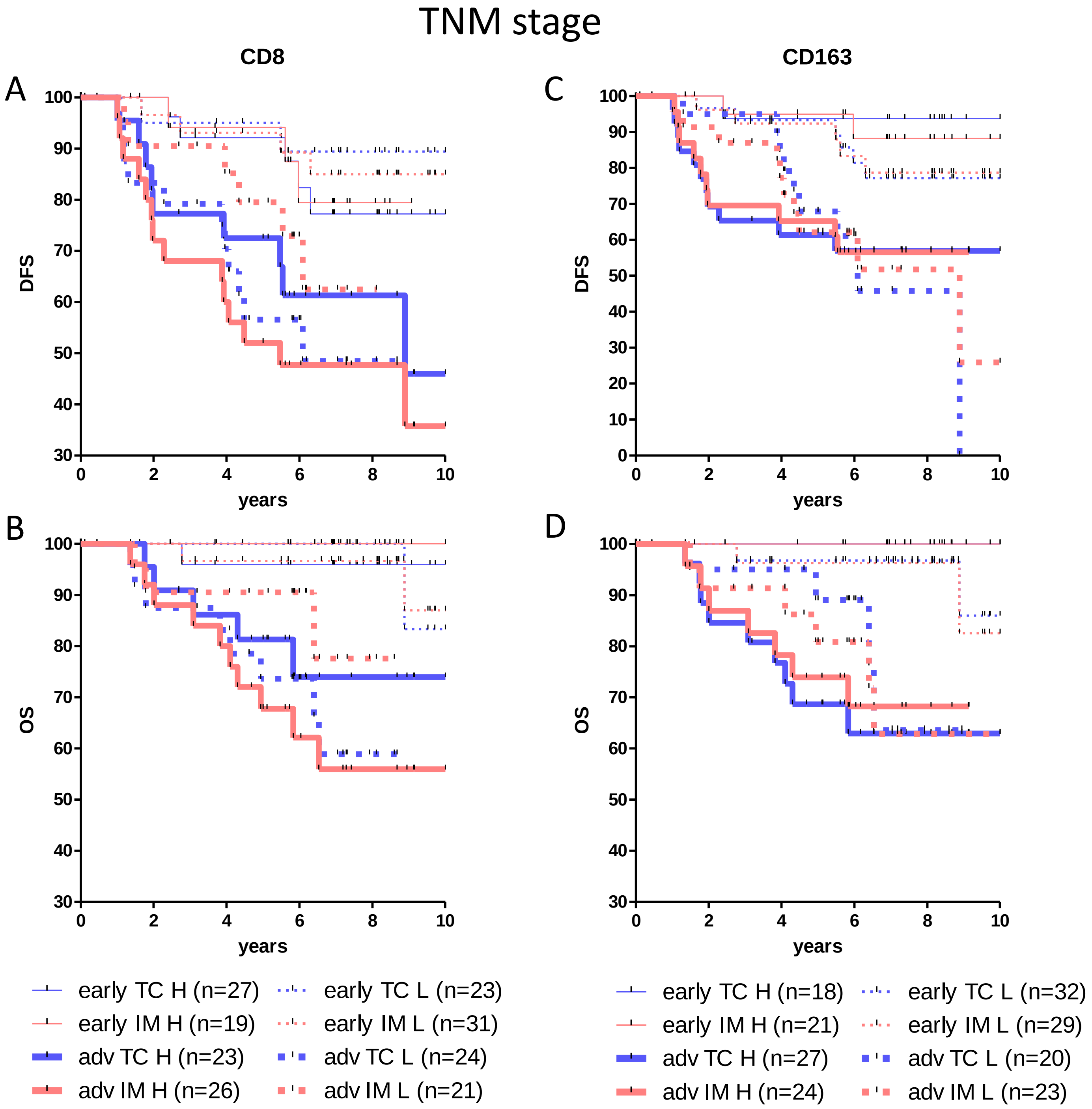

TNM stage
CD8
CD163
A
B
C
D
DFS
OS
years
100
90
80
70
60
50
40
30
20
10
0
2
4
6
8
10
early TC H (n=27)
early TC L (n=23)
early IM H (n=19)
early IM L (n=31)
adv TC H (n=23)
adv TC L (n=24)
adv IM H (n=26)
adv IM L (n=21)
early TC H (n=18)
early TC L (n=32)
early IM H (n=21)
early IM L (n=29)
adv TC H (n=27)
adv TC L (n=20)
adv IM H (n=24)
adv IM L (n=23)

Sup. Fig. 6

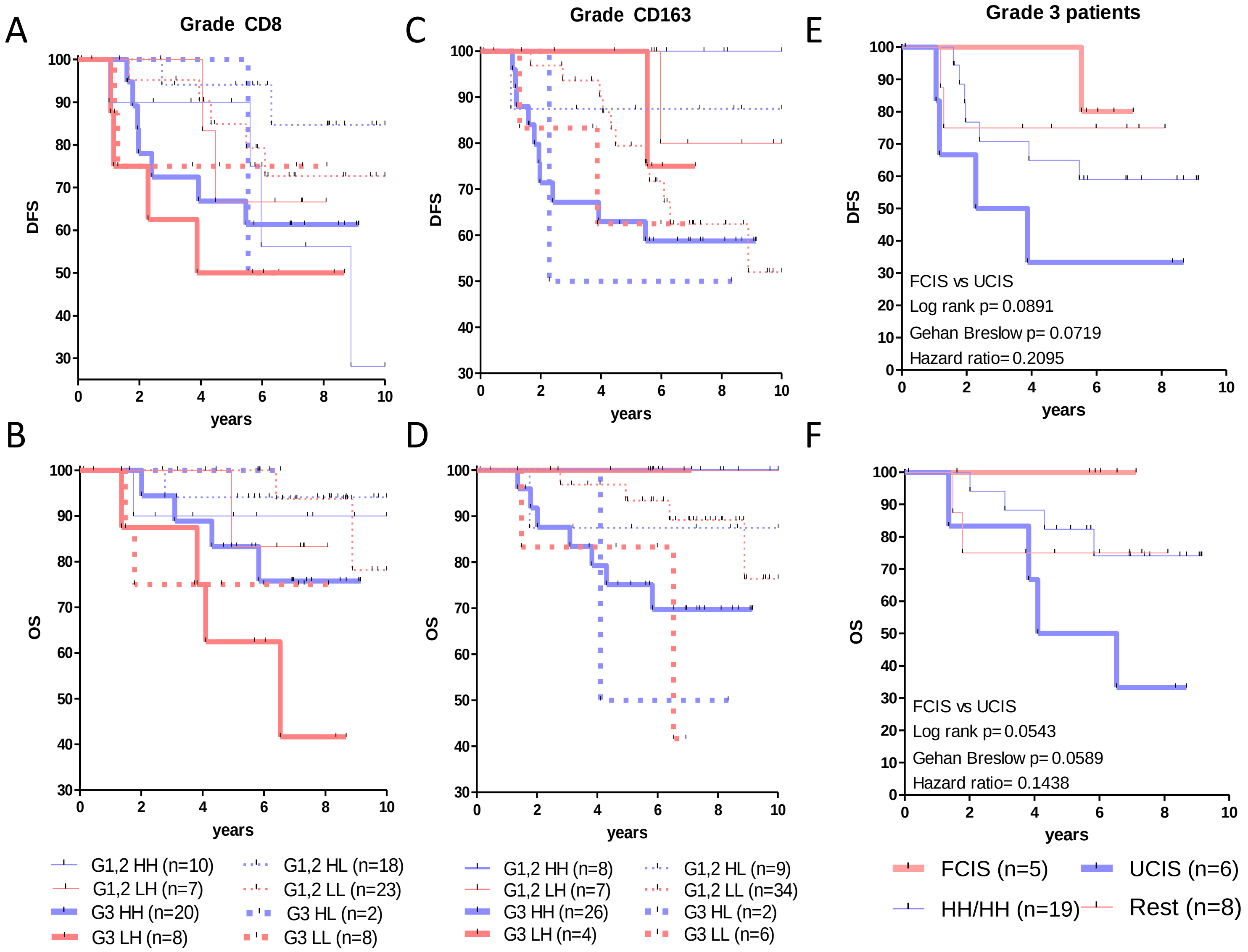

A
Grade CD8
DFS
years
B
OS
years
G1,2 HH (n=10)
G1,2 LH (n=7)
G3 HH (n=20)
G3 LH (n=8)
G1,2 HL (n=18)
G1,2 LL (n=23)
G3 HL (n=2)
G3 LL (n=8)
C
Grade CD163
DFS
years
D
OS
years
G1,2 HH (n=8)
G1,2 LH (n=7)
G3 HH (n=26)
G3 LH (n=4)
G1,2 HL (n=9)
G1,2 LL (n=34)
G3 HL (n=2)
G3 LL (n=6)
E
Grade 3 patients
DFS
FCIS vs UCIS
Log rank p= 0.0891
Gehan Breslow p= 0.0719
Hazard ratio= 0.2095
years
F
OS
FCIS vs UCIS
Log rank p= 0.0543
Gehan Breslow p= 0.0589
Hazard ratio= 0.1438
years
FCIS (n=5)
UCIS (n=6)
HH/HH (n=19)
Rest (n=8)

Sup. Fig. 7

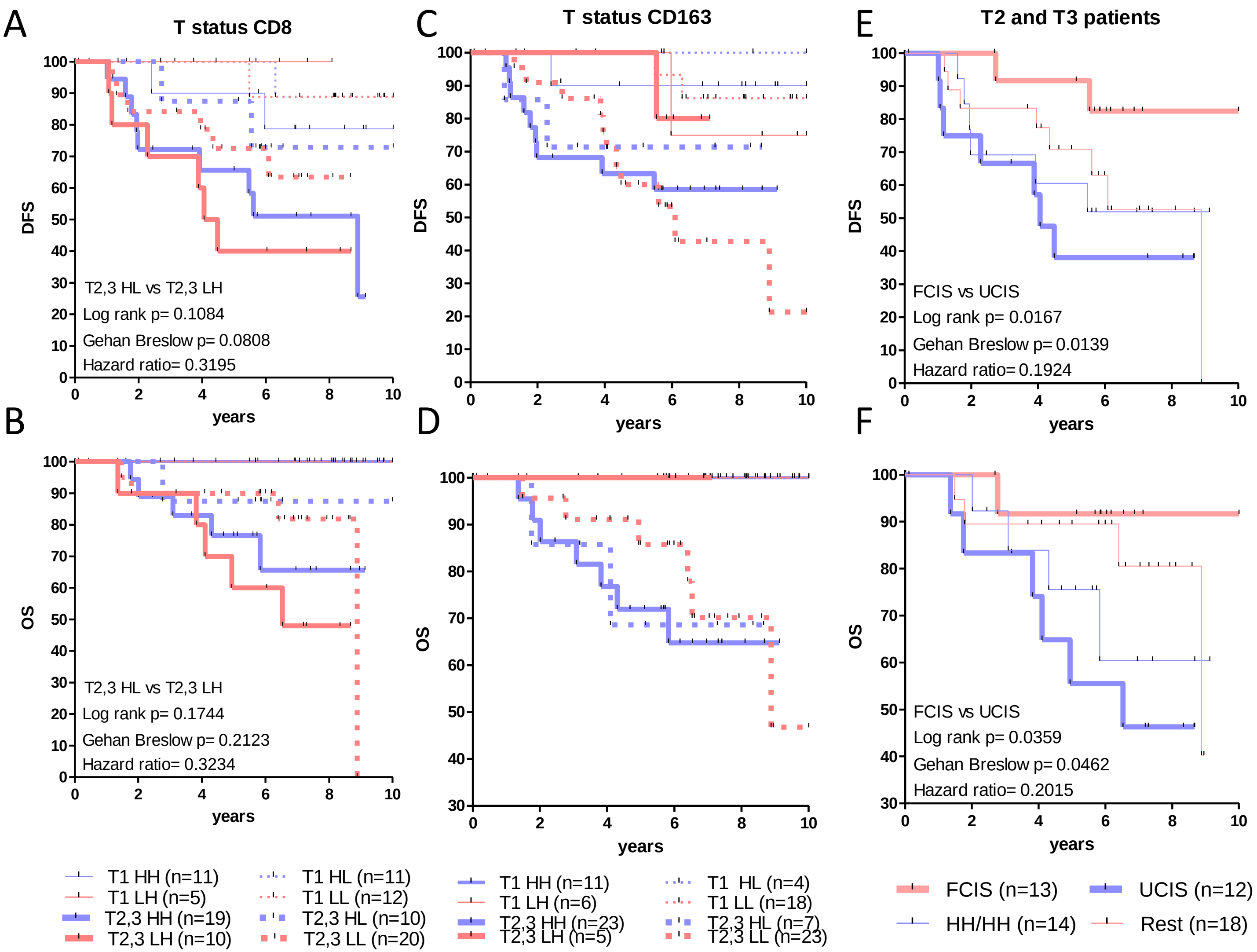
A
T status CD8
DFS
years
T2,3 HL vs T2,3 LH
Log rank p= 0.1084
Gehan Breslow p= 0.0808
Hazard ratio= 0.3195
B
OS
years
T2,3 HL vs T2,3 LH
Log rank p= 0.1744
Gehan Breslow p= 0.2123
Hazard ratio= 0.3234
T1 HH (n=11)
T1 LH (n=5)
T2,3 HH (n=19)
T2,3 LH (n=10)
T1 HL (n=11)
T1 LL (n=12)
T2,3 HL (n=10)
T2,3 LL (n=20)
C
T status CD163
DFS
years
D
OS
years
T1 HH (n=11)
T1 LH (n=6)
T2,3 HH (n=23)
T2,3 LH (n=5)
T1 HL (n=4)
T1 LL (n=18)
T2,3 HL (n=7)
T2,3 LL (n=23)
E
T2 and T3 patients
DFS
years
FCIS vs UCIS
Log rank p= 0.0167
Gehan Breslow p= 0.0139
Hazard ratio= 0.1924
F
OS
years
FCIS vs UCIS
Log rank p= 0.0359
Gehan Breslow p= 0.0462
Hazard ratio= 0.2015
FCIS (n=13)
UCIS (n=12)
HH/HH (n=14)
Rest (n=18)

Sup. Fig. 8

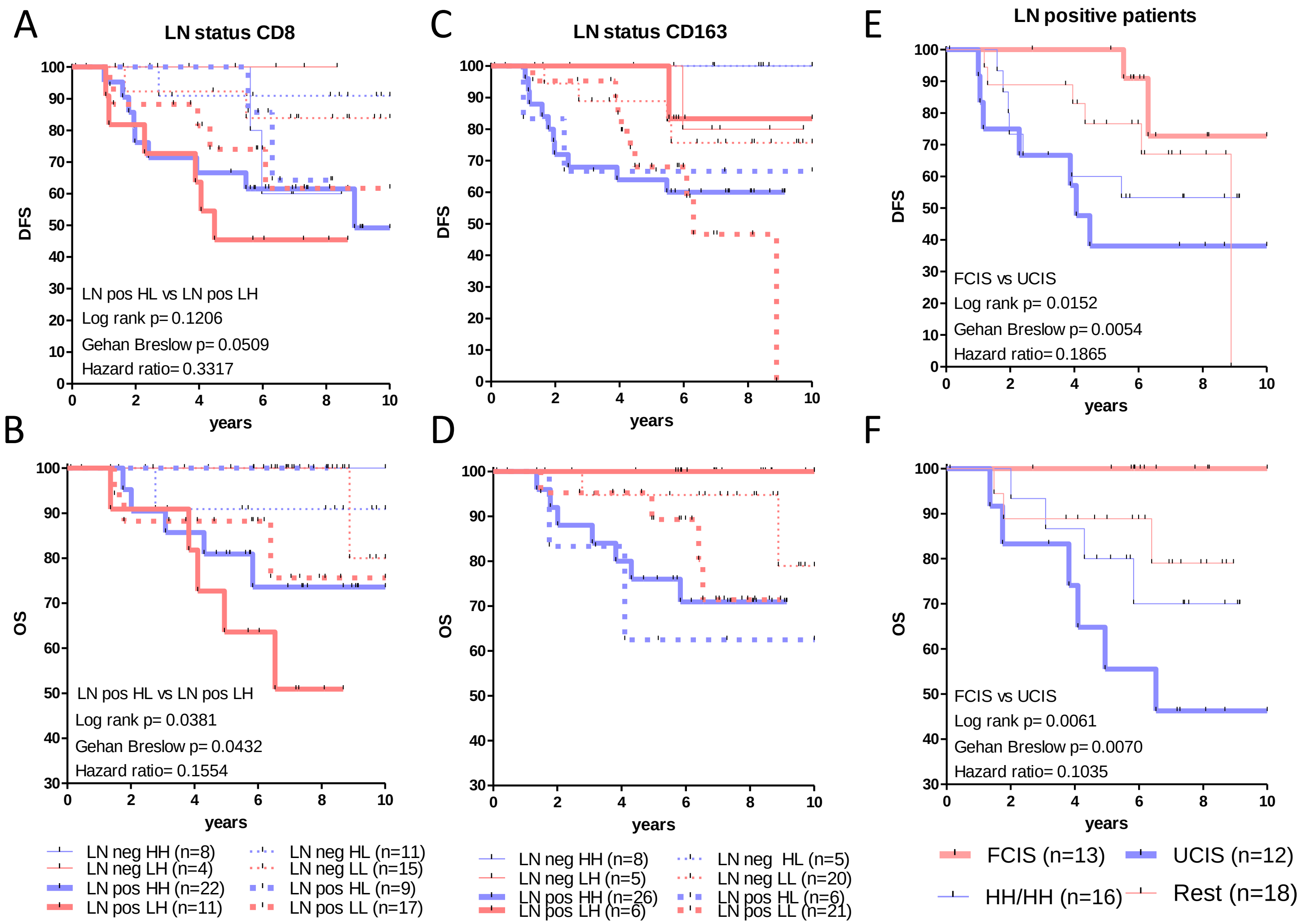

A
LN status CD8
DFS
years
LN pos HL vs LN pos LH
Log rank p= 0.1206
Gehan Breslow p= 0.0509
Hazard ratio= 0.3317
B
OS
years
LN pos HL vs LN pos LH
Log rank p= 0.0381
Gehan Breslow p= 0.0432
Hazard ratio= 0.1554
LN neg HH (n=8)
LN neg LH (n=4)
LN pos HH (n=22)
LN pos LH (n=11)
LN neg HL (n=11)
LN neg LL (n=15)
LN pos HL (n=9)
LN pos LL (n=17)
C
LN status CD163
DFS
years
D
OS
years
LN neg HH (n=8)
LN neg LH (n=5)
LN pos HH (n=26)
LN pos LH (n=6)
LN neg HL (n=5)
LN neg LL (n=20)
LN pos HL (n=6)
LN pos LL (n=21)
E
LN positive patients
DFS
years
FCIS vs UCIS
Log rank p= 0.0152
Gehan Breslow p= 0.0054
Hazard ratio= 0.1865
F
OS
years
FCIS vs UCIS
Log rank p= 0.0061
Gehan Breslow p= 0.0070
Hazard ratio= 0.1035
FCIS (n=13)
UCIS (n=12)
HH/HH (n=16)
Rest (n=18)

Sup. Fig. 9

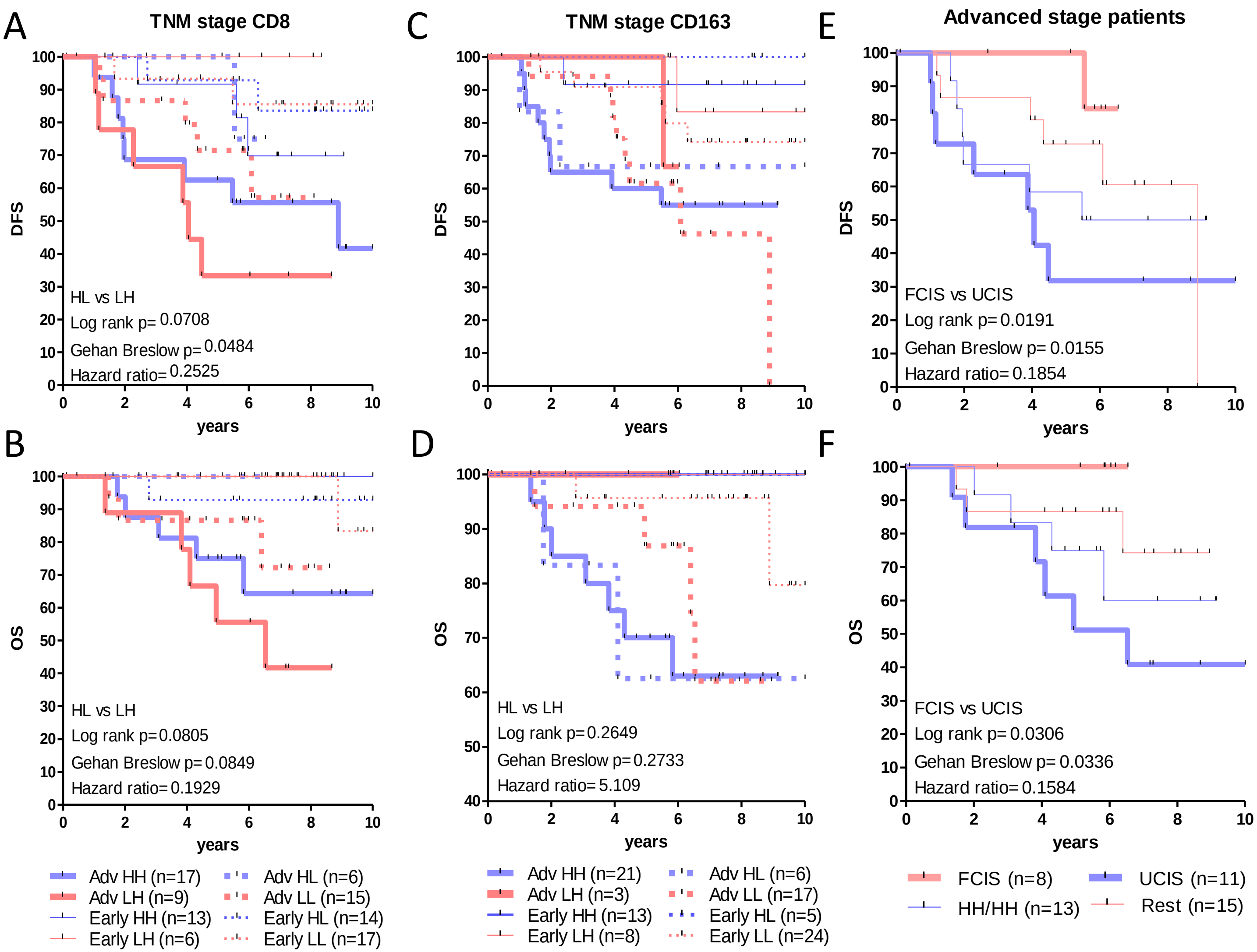
A
TNM stage CD8
DFS
years
HL vs LH
Log rank p= 0.0708
Gehan Breslow p= 0.0484
Hazard ratio= 0.2525
B
OS
years
HL vs LH
Log rank p=0.0805
Gehan Breslow p=0.0849
Hazard ratio=0.1929
Adv HH (n=17)
Adv LH (n=9)
Early HH (n=13)
Early LH (n=6)
Adv HL (n=6)
Adv LL (n=15)
Early HL (n=14)
Early LL (n=17)
C
TNM stage CD163
DFS
years
D
OS
years
HL vs LH
Log rank p=0.2649
Gehan Breslow p=0.2733
Hazard ratio= 5.109
Adv HH (n=21)
Adv LH (n=3)
Early HH (n=13)
Early LH (n=8)
Adv HL (n=6)
Adv LL (n=17)
Early HL (n=5)
Early LL (n=24)
E
Advanced stage patients
DFS
years
FCIS vs UCIS
Log rank p= 0.0191
Gehan Breslow p= 0.0155
Hazard ratio= 0.1854
F
OS
years
FCIS vs UCIS
Log rank p= 0.0306
Gehan Breslow p= 0.0336
Hazard ratio= 0.1584
FCIS (n=8)
HH/HH (n=13)
UCIS (n=11)
Rest (n=15)
